# Diabetic ketoacidosis amongst patients with COVID‐19: A retrospective chart review of 220 patients in Pakistan

**DOI:** 10.1002/edm2.331

**Published:** 2022-02-24

**Authors:** Asim Muhammad, Muhammad Hakim, Saima Afaq, Farhad Ali Khattak, Najmush Shakireen, Muhammad Jawad, Rabia Saeed, Zia Ul Haq

**Affiliations:** ^1^ Department of Medicine North West General Hospital Peshawar Pakistan; ^2^ Institute of Public Health and Social Sciences Khyber Medical University Peshawar Pakistan; ^3^ 194863 Department of Epidemiology and Biostatistics Imperial College London London UK; ^4^ Research & Development Cell Khyber College of Dentistry (KCD) Peshawar Pakistan; ^5^ Department of Pulmonology Combined Military Hospital Peshawar Pakistan; ^6^ Department of Family Medicine Khyber Medical University Peshawar Peshawar Pakistan; ^7^ Department of Medicine, Resident Internal Medicine North West General Hospital Peshawar Pakistan; ^8^ Vice Chancellor & Dean Khyber Medical University Peshawar Peshawar Pakistan

**Keywords:** COVID‐19, diabetes mellitus, diabetic ketoacidosis

## Abstract

**Objectives:**

To determine the frequency of diabetes mellitus and diabetic ketoacidosis and associated factors in COVID‐19‐positive patients.

**Background:**

High mortality amongst SARS‐Cov2 patients may be attributed to diabetes and diabetic ketoacidosis.

**Methods:**

A total of 220 COVID‐19 positive patients, hospitalized in North West General Hospital & Research Center, Peshawar, KP, Pakistan, from April to September 2020, were analysed using STATA 14. Patients with positive PCR were labelled as COVID‐19 positive and were included in the study. Patients with a clinical picture of COVID‐19 and negative PCR were excluded from the study. Those having ketonemia >0.6 and random blood glucose level >250mg/dl, while HCO3 (bicarbonate) ≤18, were labelled as diabetic ketoacidosis. The statistical significance level was set at *p* < .05.

**Results:**

A total of 220 COVID‐19 patients were admitted; 166 (75.4%) were male and 54 (24.5%) were female. The mean age in years of the patients was 55.95 (SD13.9). About 57.7% of patients had diabetes mellitus, and 15 (6.8%) patients developed diabetic ketoacidosis. Amongst those with DKA, 5 patients died during hospital admission. The use of steroids was significantly higher (*p *< .001) in the DKA group compared with non‐DKA patients. Hypertension (103,46.8%) and fever (170,77.3%) were the most reported comorbidity and symptom respectively.

**Conclusion:**

The proportion of diabetes mellitus is high in patients with COVID‐19. Diabetic ketoacidosis is a frequent complication in this group associated with in‐hospital mortality. Steroid administration for COVID‐19 should be balanced with strict glycemic control to prevent diabetic ketoacidosis and increase hospital survival.

## Abstract 2

The newly diagnosed diabetes mellitus as a result of metabolic disturbances from COVID‐19 illnesses can lead to diabetic ketoacidosis (DKA). Our data showed that 6.82% of patients developed diabetic ketoacidosis. Usage of steroids was significantly higher while hypertension and fever were the most common reported comorbidity. We concluded that the incidence of diabetes mellitus is high in patients with COVID‐19. Diabetic ketoacidosis is a frequent complication associated with in‐hospital mortality. Steroid administration for COVID‐19 should be balanced with strict glycemic control to prevent diabetic ketoacidosis and increase hospital survival.

## 1 INTRODUCTION

Coronavirus disease 2019 (COVID‐19) has become a catastrophic pandemic affecting people throughout the world and severely disturbing public health security.^1^, ^2^ Its pathogen, severe acute respiratory syndrome coronavirus 2 (SARS‐CoV‐2), is the third identified human beta‐coronavirus, which is reported to target pulmonary systems^3^, ^4^Clinical spectrums of COVID‐19 vary from asymptomatic to lethal pneumonia.^5^ Besides these, COVID‐19 has unpredictable effects on many organs. However, data regarding the endocrine impact of COVID‐19 are limited.

Rubino et al^6^ proposed that SARS‐CoV‐2 leads to ketosis‐prone diabetes via binding to its cellular entry ACE‐2 receptors, which are abundant in pancreatic beta cells and adipose tissue, leading to glucose metabolism abnormalities and pancreatic beta‐cell destruction. This mechanism underlies the development of diabetes mellitus (DM) in SARS‐CoV2 patients.^7^ Moreover, SARS‐CoV‐2 may induce an autoimmune attack on the pancreatic islet cells mimicking the pathogenesis of insulin‐dependent DM.^8^ The elevated HbA1C and the presence of DM risk factors in several patients may indicate that the newly diagnosed DM is a result of metabolic disturbances from COVID‐19 illnesses unmasking the existing DM^9^ rather than causing the new onset of disease^10^, ^11^ However, the unusual high incidences of diabetic ketoacidosis (DKA) in type 2 DM raise the issue of whether COVID‐19 can further damage pancreatic islet cells leading to insulin deficiency states.^12^, ^13^

The present study aimed to determine the frequency of DKA and diabetes mellitus in COVID‐19 patients and compare the clinical characteristics and associated factors of patients with non‐DKA to those with DKA.

## 2 METHODS

This retrospective chart review was carried out in the COVID‐19 ward in Northwest General Hospital & Research Center Peshawar, Pakistan, which is a tertiary care hospital. Records of patients diagnosed with COVID‐19, from 1 April 2020 to 30 September 2020, were obtained from the hospital. Patient records are stored on encrypted online servers and regularly updated by the on‐duty doctors. Approval from Independent Ethical Committee (NWGH/2378) was obtained to access the data from the hospital server system and use it for the current research. All laboratory investigations were done during the patient admission in the hospital laboratory. Nasopharyngeal swabs were taken for confirmation of SARS COV‐2 through PCR. Patients with positive PCR were labelled as COVID‐19 positive and were included in the study. Clinically suspected COVID‐19 patients with negative PCR while those who had positive PCR but refused in‐hospital treatment were excluded. Patients with a history of diabetes, with a confirmed physician's diagnosis, or who were on a specific diet and/or were already taking oral hypoglycemic or insulin, were labelled as previously known diabetes, while patients with no history of diabetes before admission and had HbA1c of equal to or more than 6.5% done during this admission were labelled as previously unknown diabetes. Patients with all three of the following were labelled as diabetic ketoacidosis (DKA).

1. Ketonemia >0.6
2. Random blood glucose level >250 mg/dl
3. HCO3 (bicarbonate) ≤18^14^

Data were analysed using STATA 14. Continuous variables are presented as mean (SD) while categorical variables as percentages and numbers. Frequencies of demographic characteristics, symptoms and comorbidities were described and compared between the survivors and no survivors using the chi‐squared and Fisher's exact test. The statistical significance level was set at *p* < .05 (two‐sided).

## 3 RESULTS

Table 1 summarizes the demographic and clinical characteristics of the patients. A total of 220 patients were diagnosed with COVID‐19 and admitted to the NWGH between 1 April and 30 September 2020, and their data were analysed. Amongst them, 166 (75.4%) were male and 54 (24.5%) were female. The mean age in years of the patients was 55.9 (SD 13.90), while 88 (40%) patients were in the age category 56–72 years. The mean duration of stay at the hospital (in days) was 7.83 (SD 7.3). The mean duration of symptoms in days was 7.9 (SD 5.44) with approximately 78% of the patients recovering from symptoms within 10 days.

**TABLE 1:** Demographics and baseline characteristics of all (*N* = 220) patients with COVID‐19

Demographic variable (*N* = 220)	*N*	%
Age categories		
22–38 Years	27	12.3
39–55 Years	76	34.5
56–72 Years	88	40
73–89 Years	29	13.2
Length of stay in hospital		
1–10 Days	169	76.8
11–20 Days	42	19.1
21–30 Days	6	2.7
31–40 Days	1	0.4
41–50 Days	1	0.4
51–60 Days	1	0.4
Duration of symptoms		
0–10 Days	172	78.2
11–20 Days	42	19.1
21–30 Days	6	2.8
Gender		
Male	166	75.5
Female	54	24.6
Health status outcome		
Recovered	168	76.3
Died	52	23.6
Status of DM		
Non‐DM	93	42.2
Previously known DM	86	39.1
Previously unknown DM	41	18.6
Status of DKA		
Non‐DKA	205	93.1
DKA	15	6.8
Comorbidities		
HTN	103	46.8
CAD	34	15.4
CCF	6	2.7
CKD	12	5.4
COPD	5	2.2
Asthma	23	10.4
Arrhythmias	6	2.7
Symptoms		
Cough	146	66.3
Fever	170	77.2
Sputum	14	6.3
Body aches	59	26.8
Sore throat	11	5
SOB	145	65.9
Chest pain	26	11.8
Headache	8	3.6
Diarrhoea	13	5.9
Nausea/Vomiting	7	3.1
Myalgias	1	0.4
Clinical management		
Ionotropic support	24	10.9
NIV support	76	34.5
Invasive mechanical ventilation	29	13.1
Tociluzimab	89	40.4
HDU care	33	15
ICU care	46	20.9
Use of steroids	80	36.3

**FIGURE 1:**
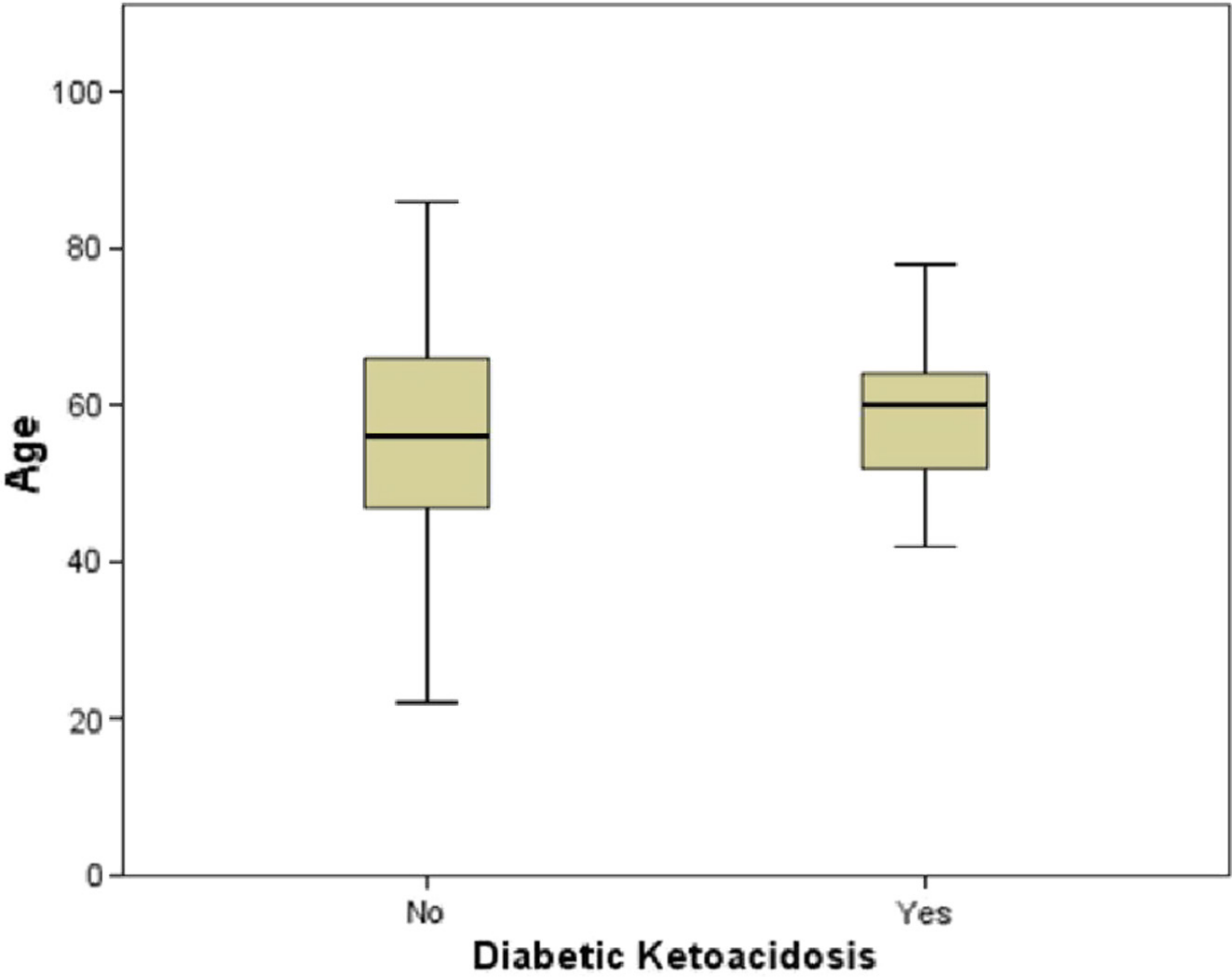
Box plot for age distribution amongst non‐DKA patients and those with DKA

Of 220 COVID‐19 diagnosed patients, 52 (23.6%) died during hospital admission. The proportion of previously known and previously unknown diabetics was 39.1% and 18.6% respectively. We identified 15 (6.8%) patients with DKA, based on the biochemical levels of ketones, blood glucose and HCO3 (bicarbonate). The mean age of those newly diagnosed with DKA was identified as 57.7 (SD12.0) (Figure 1). Hypertension was the most reported comorbidity in 103 (46.8%) patients, and amongst different symptoms, fever was the most reported in 170 (77.3%). Regarding clinical management, a total of 46 (20.9%) patients were treated in ICU. Tocilizumab and steroid were received by 89 (40.4%) and 80 (36.4%) patients respectively.

A total of 15 (6.8%) COVID‐19 patients were diagnosed with DKA. Amongst these, 10 (66.7%) were males and 5 (33.3%) were females. Out of the 15 DKA patients, 9 were aged above 55 years while 6 were less than 55 years of age. The number of DKA patients who died and recovered was 5 (33.3%) and 10 (66.7%) respectively. Regarding the status of diabetes mellitus of DKA patients, 11(73.3%) had previously known diabetes and 4 (26.6%) had previously unknown diabetes. (Table 2).

**TABLE 2:** Demographic details of patients with status of non‐DKA vs. DKA

Variables	Categories	Status of DKA			
		Non‐DKAN = 205%–93.18%	DKA *N* = 15–6.82%	Total:220%–100%	*p*‐Value
Age (In years)	22–38 Years	26 (12.6)	1 (6.6)	27 (12.0)	0.6
	39–55 Years	71 (34.6)	5 (33.)	76 (34.5)	
	56–72 Years	80 (39.0)	8 (53.3)	88 (40.0)	
	73–89 Years	28 (13.6)	1 (6.6)	29 (13.1)	
Gender	Male	156 (76.1)	10 (66.6)	166 (75.4)	0.4
	Female	49 (23.9)	5 (33.3)	54 (24.5)	
Clinical outcome	Recovered	158 (77.0)	10 (66.6)	168 (76.3)	0.3
	Died	47 (22.9)	5 (33.3)	52 (23.6)	
Status of DM	No	93 (45.3)	0 (0.00)	93 (42.2)	0.01
	Previously known DM	75 (36.5)	11 (73.3)	86 (39.0)	
	Previously unknown DM	37 (18.0)	4 (26.6)	41 (18.6)	

Diabetic Ketoacidosis patients with hypertension was present in 5 (33.3%) patients and a similar number required management in ICU. The proportion of DKA patients who received steroids (86.7%) was significantly higher (*p *< .001) than that of non‐DKA patients (32.7%). There were no significant differences in other demographic and clinical characteristics between patients with DKA and those without DKA (non‐DKA). (Tables 3 and 4).

**TABLE 3:** Clinical characteristics of patients with status of non‐DKA vs. DKA

Variables	Categories	Status of DKA			
		Non‐DKA *N* = 205 93.18%	DKA *N* = 15 (6.82%)	Total 220 (100%)	*p*‐Value
Status of HTN	Yes	98 (47.8)	5 (33.3)	103 (46.8)	0.3
Status of CAD	Yes	30 (14.6)	4 (26.7)	34 (15.4)	0.2
Status of CCF	Yes	05 (2.4)	01 (6.6)	6 (2.73)	0.3
Status of CKD	Yes	12 (5.8)	0 (0.00)	12 (5.5)	0.3
Status of COPD	Yes	04 (1.9)	01 (6.7)	05 (2.3)	0.7
Status of asthma	Yes	21 (10.2)	2 (13.3)	23 (10.4)	0.7
Status of arrhythmias	Yes	06 (2.9)	0 (0.00)	06 (2.7)	0.5

**TABLE 4:** Clinical management of patients with status of non‐DKA vs. DKA

Variables	Categories	Status of DKA			
		Non‐DKA *N* = 205 (93.18%)	DKA *N* = 15 (6.82%)	Total 220 (100%)	*p*‐Value
Ionotropic support	Yes	23 (11.2)	1 (6.7)	24 (10.9)	0.58
NIV support	Yes	70 (34.1)	6 (40.0)	76 (34.5)	0.64
Invasive mechanical ventilation	Yes	27 (13.1)	2 (13.3)	29 (13.2)	0.99
High Dependency Unit (HDU)	Yes	29 (14.1)	4 (26.6)	33 (15.0)	0.19
ICU care	Yes	41 (20.0)	5 (33.3)	46 (20.9)	0.22
Steroids	Yes	67 (32.6)	13 (86.6)	80 (36.3)	<.001
Tociluzimab	Yes	83 (40.4)	6 (40.0)	89 (40.4)	0.97

## 4 DISCUSSION

The objective of our study was to determine the frequency of COVID‐19 patients having DKA and compare the clinical characteristics and associated factors of patients with DKA to those without DKA. Our results show that most of the participants were males, and a major proportion was aged above 56 years while the mean length of the hospital was ~8 days. Amongst the total 220 COVID‐19 patients, almost 6.82% was newly diagnosed, whereas almost 26.6% was previously known patients of diabetes mellitus. Hypertension was the most common comorbidity overall as well as amongst the patients with DKA. Comparison of biochemical and inflammatory parameters between patients with DKA and those without DKA showed that HbA1c, ketones, blood glucose and urea were higher in the former. In contrast, HC03 and HB levels were lower in patients with DKA compared to patients without DKA while the other biochemical markers and all inflammatory markers were similar between the two groups. The mortality in non‐DKA was 22.1% while in DKA is high, 33.4%.

There is a greater risk for the previously unknown diabetes mellitus suffering from COVID‐19.^15^ Diabetic ketoacidosis is a lethal complication of severe COVID‐19 with poor prognostic outcomes.^16^ In our study, 6.9% was diagnosed with DKA, and many of them were unaware of their diabetes mellitus status. A case report by Nadine E Palermo found a 53 years woman who presented to the emergency department in Boston with elevated biochemical cytokines, but she had no diabetes‐related complications before her admission and was started IV insulin with the supportive treatment of ketoacidosis.^17^ The reason whether COVID‐19 can damage pancreatic islet cells, leading to insulin deficiency, disturbed glucose homeostasis, inflammation, altered immune status and activation of renin–angiotensin–aldosterone system.^12^

In our study, hypertension was the most reported comorbidity amongst all. A meta‐analysis by Bianca de Almeida‐Pititto showed that OR was 2.98 for an association of hypertension with COVID‐19 infection through random effect model.^18^ Similarly, a study conducted by Sun et al showed hypertension was associated with an increased risk of severe COVID‐19 infection.^18^, ^19^ Along with high HbAIc in patients with DKA, ketones, blood glucose, urea and Hb levels were deranged, compared to normal reference values, in our study. A similar study by Juyi Li found that patients with diabetic ketosis developed acidosis, amongst whom 26.7% died.^20^ This suggests that COVID‐19 infection caused ketosis or ketoacidosis while induced diabetic ketoacidosis for those with diabetes. Likewise, ketosis leads to the length of hospital stay and mortality.^21^ In our study, the mean length of the hospital was eight days. The results of this study showed that the mortality rate in non‐DKA patients was 22.9%, while in patients with DKA, it was 33.3%. Furthermore, in a study conducted in Saudi Arabia, 4 patients were recovered and discharged to their homes and 1 had a complicated course and died,^22^ which is contradictive to our result.

The strengths of our study are: (i) no previous study has been conducted on this topic in Pakistan and (ii) the biochemical and inflammatory markers were done for all the patients. However, this study was conducted in a single private tertiary care hospital; therefore, caution may be taken before generalizing the results for other populations. Moreover, due to the system constraints, arterial blood gases were not done for all the patients.

## 5 CONCLUSIONS

COVID‐19 likely unmasked existing undiagnosed diabetes by provoking its metabolic complications. Both diabetes and DKA affect COVID‐19 outcomes and aggravate mortality. Those diagnosed with DKA were mostly ignorant of their diabetes status. The inter‐relationship between diabetes and COVID‐19 should prompt more research to understand the extent to which specific mechanisms of the virus might add to the deteriorating glycemic control and, in some cases, to the striking development of diabetic ketoacidosis. More research is necessary to investigate the causal relationship between diabetes, DKA and COVID‐19.

## CONFLICT OF INTEREST

None to declare.

## AUTHOR CONTRIBUTIONS

**Asim Muhammad:** Conceptualization (equal); Data curation (equal); Software (lead). **Muhammad Hakim:** Formal analysis (lead); Investigation (equal); Writing – original draft (equal). **Saima Afaq:** Formal analysis (equal); Writing – original draft (equal). **Farhad Ali Khattak:** Formal analysis (equal); Writing – original draft (equal). **Najmush Shakireen:** Data curation (equal); Investigation (equal). **Muhammad Jawad:** Data curation (equal); Project administration (equal). **Rabia Saeed:** Data curation (equal); Investigation (equal). **Zia Ul Haq:** Project administration (equal); Writing – review & editing (equal).

## ETHICAL APPROVAL

The study was approved by the ethical committee of Northwest General Hospital Peshawar, Khyber Pakhtunkhwa, Pakistan. (NWGH/2378).

## Data Availability

The data that support the findings of this study are available in the supplementary material of this article.
